# Genome-wide association analysis reveals loci and candidate genes involved in fiber quality traits in sea island cotton (*Gossypium barbadense*)

**DOI:** 10.1186/s12870-020-02502-4

**Published:** 2020-06-22

**Authors:** Xiujuan Su, Guozhong Zhu, Xiaohui Song, Haijiang Xu, Weixi Li, Xinzhu Ning, Quanjia Chen, Wangzhen Guo

**Affiliations:** 1 State Key Laboratory of Crop Genetics and Germplasm Enhancement, Engineering Research Center of Hybrid Cotton Development (the Ministry of Education), Nanjing Agricultural University, Nanjing, 210095 China; 2 Engineering Research Center for Cotton (the Ministry of Education), Xinjiang Agricultural University, Urumqi, 830052 China; 3 Institute of Industrial Crops, Xinjiang Academy of Agricultural Sciences, Urumqi, 830091 China; 4 Cotton Research Institute, Xinjiang Academy of Agricultural and Reclamation Science, Shihezi, 832000 China

**Keywords:** *Gossypium barbadense*, Genetic diversity, Genome-wide association study, Fiber quality, Quantitative trait loci, Salt stress

## Abstract

**Background:**

Sea island cotton (*Gossypium barbadense*) has markedly superior high quality fibers, which plays an important role in the textile industry and acts as a donor for upland cotton (*G. hirsutum*) fiber quality improvement. The genetic characteristics analysis and the identification of key genes will be helpful to understand the mechanism of fiber development and breeding utilization in sea island cotton.

**Results:**

In this study, 279 sea island cotton accessions were collected from different origins for genotyping and phenotyping fiber quality traits. A set of 6303 high quality single nucleotide polymorphisms (SNPs) were obtained by high-density CottonSNP80K array. The population characteristics showed that the sea island cotton accessions had wide genetic diversity and were clustered into three groups, with Group1 closely related to Menoufi, an original sea island cotton landrace, and Group2 and Group3 related to widely introduced accessions from Egypt, USA and Former Soviet Union. Further, we used 249 accessions and evaluated five fiber quality traits under normal and salt environments over 2 years. Except for fiber uniformity (FU), fiber length (FL) and fiber elongation (FE) were significantly decreased in salt conditions, while fiber strength (FS) and fiber micronaire (MIC) were increased. Based on 6303 SNPs and genome-wide association study (GWAS) analysis, a total of 34 stable quantitative trait loci (QTLs) were identified for the five fiber quality traits with 25 detected simultaneously under normal and salt environments. Gene Ontology (GO) analysis indicated that candidate genes in the 25 overlapped QTLs were enriched mostly in “cellular and biological process”. In addition, “xylem development” and “response to hormone” pathways were also found. Haplotype analyses found that *GB_A03G0335* encoding an E3 ubiquitin-protein ligase in QTL TM6004 had SNP variation (A/C) in gene region, was significantly correlated with FL, FS, FU, and FE, implying a crucial role in fiber quality.

**Conclusions:**

The present study provides a foundation for genetic diversity of sea island cotton accessions and will contribute to fiber quality improvement in breeding practice.

## Background

Cotton (*Gossypium* spp.) is one of the most important crops producing natural fibers for the textile industry. Upland cotton (*G. hirsutum*) and sea island cotton (*G. barbadense*) are the two main cultivated tetraploid species accounting for more than 97% of the total cotton production [1]. Upland cotton produces high yields with moderate fiber quality, whereas sea island cotton has exceptionally high quality fibers [2]. As the textile industry rapidly develops and the demand for textile products continues to diversify, the improvement of cotton fiber quality is more and more important [3]. The introgression of chromosome segments can effectively combine the yield advantage of upland cotton with the quality improvement from sea island cotton [4]. Hence, it is of great significance to explore the elite chromosome segments in *G. barbadense* for improving cotton fiber quality in breeding.

Identifying the quantitative trait loci (QTLs) of the target traits and marker assisted selection (MAS) greatly accelerated the breeding of cotton varieties. With the development of molecular marker, SNP array and resequencing technology, a batch of QTLs for fiber yield, quality and salt tolerance of upland cotton have been explored. Using SSR markers, Shen et al. (2005) identified 39 QTLs associated with fiber quality traits of upland cotton [5]. Sun et al. (2018) genotyped 719 upland cotton germplasm accessions by an Illumina CottonSNP63K array and 62 SNP loci were significantly associated with yield-related traits [6]. Via specific-locus amplified fragment sequencing (SLAF-seq), Su et al. (2016) detected 12 SNPs associated with lint percentage of upland cotton in four different environments [7]. For salt tolerance, 8 and 23 SNPs were identified to be associated significantly with the salt-tolerance related traits [8, 9]. These studies provided QTLs and gene resources for developing upland cotton varieties with high yields and salt stress tolerance.

Compared with *G. hirsutum*, less studies for genetic diversity and QTL mining related to target traits were reported using natural population in *G. barbadense*. In addition, Xinjiang is the main production area of sea island cotton in China, however, most of the cultivated land in Xinjiang is saline alkali land [10]. Salinity is a serious threat for fiber yield and quality [11]. In upland cotton, higher Na^+^ levels in soil can decrease fiber length, strength, and micronaire [12]. However, the related report on sea island cotton is rare. Based on these, in this study, 279 sea island cotton accessions were collected from different origins, and genotyping for population structure and phenotyping for fiber qualities in different soil environments over 2 years were systematically investigated. By combining the two datasets, genome-wide association study (GWAS) analysis identified the stable QTLs and candidate genes associated with fiber qualities. The finding will provide elite genetic resources with candidate QTLs and genes for fiber quality improvement in cotton breeding.

## Results

### Genetic variation based on SNPs

The 279 sea-island cotton accessions (Additional file 1: Table S1) were genotyped using the CottonSNP80K array. The genotypic data revealed that these cotton accessions possessed a high average call rate of 96.54% (Additional file 2: Fig. S1). We further filtered some loci with call frequency < 80%, minor allele frequency (MAF) < 0.01 or mapping to multiple loci of chromosomes). As a result, a final set of 6303 SNPs were obtained, with 3656 and 2647 SNPs on the At and Dt sub-genomes, respectively (Additional file 3: Fig. S2). To evaluate the genotyping accuracy and reproducibility, eight cotton accessions from different sources were set biological replicates for genotyping analysis. The genotyping consistencies between the replicates were ranged from 86.3 to 99.8%, indicating that there exists heterogeneity of the same accession with different sources. Of them, Hai7124 and Xinhai 26 from different sources were detected the highest consistency with 99.8 and 99.3%, respectively. We also evaluated the polymorphism information content (PIC) values of 279 accessions, which varied from 0.189 to 0.469 among chromosomes, and the mean PIC value of the At and Dt sub-genomes was 0.391 and 0.338, respectively (Additional file 4: Table S2). These results showed that the CottonSNP80K array could genotype sea island cotton accessions with good efficiency and accuracy.

### Population characteristics analysis

**Fig. 1:**
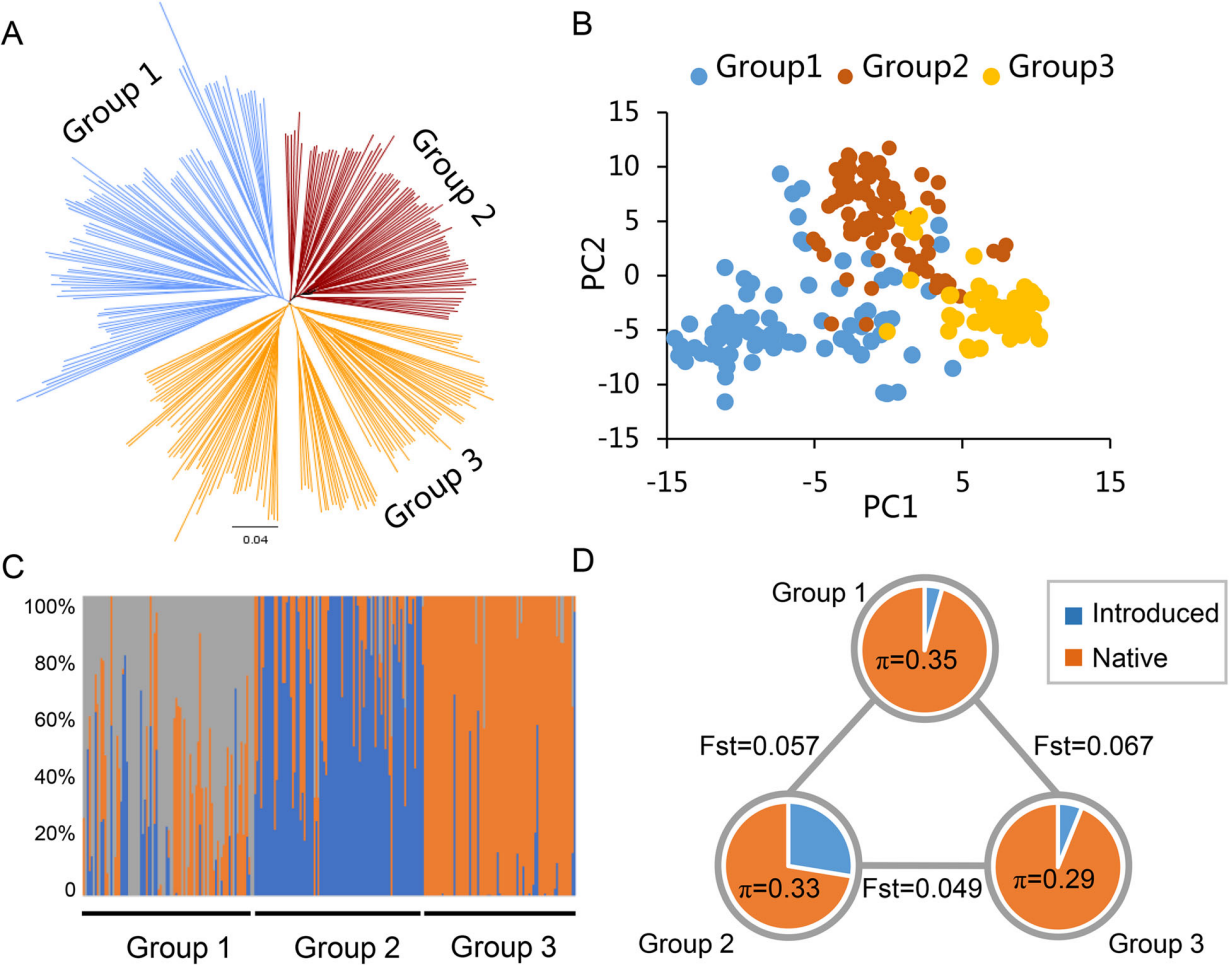
Population characteristics of the 279 sea island cotton accessions. **a**. A neighbor-joining tree was constructed using SNP data. **b**. PCA plot of the first two components (PC1 and PC2). **c**. Population structure analysis with K = 3. **d**. Nucleotide diversity (π) and population divergence (F*st*) across the three cotton groups. The pie charts show percentage of introduced accessions. The value in each circle represents nucleotide diversity for this group, and the value on each line indicates population divergence between the two groups

In order to explore the population characteristics of the sea island cotton accessions, a neighbor-joining tree was conducted using TASSEL 5.0. It showed that the 279 accessions could be clustered into three groups, which contained 87, 112 and 80 in Group1, Gourp2 and Group3, respectively (Fig. 1a). The 39 landraces, which were earlier introduced into China and contributed to sea island cotton breeding of China, were widely distributed in the three groups. Group1 contained three introduced accessions, Giza 36, Giza 81 and Menoufi, that they were all from Egypt and Menoufi was an original sea island cotton landrace. Group2 and Group3 were clustered with widely introduced accessions from Egypt, USA and Former Soviet Union, with 31 introduced accessions in Group2 and five in Group3. The PCA (Fig. 1b) and population structure analysis (Fig. 1c) also supported the 279 accessions could be clustered into three groups which agreed well with the neighbor-joining tree. We measured the genetic diversity of each group by calculating nucleotide diversity (π) values (Fig. 1d), and found that Group1 had the highest genetic diversity (π = 0.35), while Group3 was the lowest (π = 0.29). Further, population fixation statistics (*Fst*) indicated that the *Fst* value between Group1 and Group2 or between Group1 and Group3 was higher than that between Group2 and Group3 (Fig. 1d), implying that Group1 had the highest differentiation compared with the other two groups.

### Phenotypic variation in fiber quality traits

**Fig. 2:**
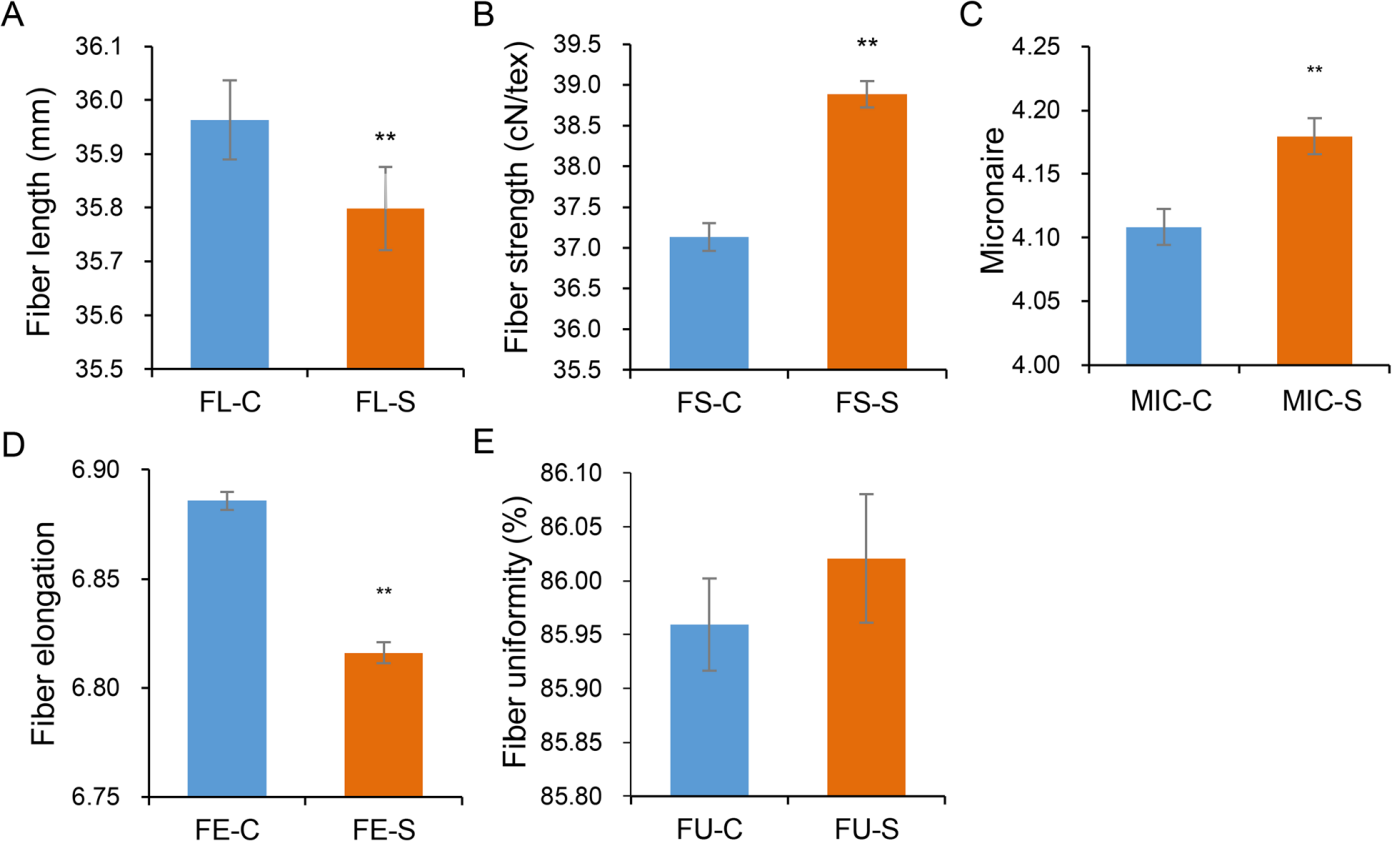
Comparison of different phenotypic data under normal and salt environments. **a**. Fiber length (FL). **b**. fiber strength (FS). **c**. fiber micronaire (MIC) **d**. fiber elongation (FE). **e**. fiber uniformity (FU). Significant difference of single trait was calculated with paired-samples *t*-tests. ** indicates a significance level of 0.01

To evaluate the phenotypic variation in fiber quality traits in the association population, we analyzed five traits in two environments over 2 years. The results showed the wide variation in the natural population with 29.35 to 39.68 mm for FL, 28.29 to 48.10 cN/tex for FS, 3.10 to 5.23 for MIC, 81.75 to 88.65% for FU and 6.52 to 7.12% for FE, respectively (Table 1). MIC and FS had the largest coefficients of variation (CV), ranging from 6.28 to 8.31% and from 8.53 to 10.91%, respectively, followed by FL (4.27–5.35%). FE and FU had the smallest CV, ranging from 1.04 to 1.48% and from 0.93 to 1.06%, respectively. Density distributions indicated that FL and FE had more high value distribution under normal conditions than under salt conditions. In details, the distribution of FL value higher than 37 and of FE value higher than 6.9 under normal conditions was more than that under salt conditions. For FS and MIC, it had higher value distribution under salt conditions than under normal conditions with greater than 32 and 4, respectively. For FU, the distribution was more concentrated under salt conditions than that under normal conditions (Additional file 5: Fig. S3). Paired-samples *t-*tests further showed that comparing to normal conditions, FL and FE decreased significantly with 0.46 and 1.01% under salt conditions, respectively, while FS and MIC increased with 4.73 and 1.74%, respectively. There was no significant difference in FU in the two environments (Fig. 2). Correlation analysis showed high phenotypic correlation for each trait under both normal and salt conditions. In addition, under the same condition, FL was significantly positively correlated with FS, FE and FU, and a positive correlation was also found among FS, FE and FU. MIC was significantly negatively correlated with FL and FS, and positively correlated with FE (Additional file 6: Fig. S4).

**Table 1:** Phenotypic variation for five fiber quality traits on 249 sea island cotton accessions

Trait	Environment^a^	Min	Max	Mean	SD	CV (%)
FL (mm)	FL17-C	31.15	39.05	36.11	1.67	4.62
	FL17-S	31.25	38.60	35.91	1.53	4.27
	FL18-C	30.31	38.45	35.82	1.62	4.52
	FL18-S	29.35	39.68	35.69	1.91	5.35
	FL-C-BLUP	25.75	41.14	35.96	3.11	8.66
	FL-S-BLUP	25.14	41.50	35.80	3.22	8.98
FS (cN/tex)	FS17-C	29.47	45.47	37.23	3.72	10.00
	FS17-S	30.90	46.30	38.07	3.25	8.53
	FS18-C	28.29	48.10	37.03	4.04	10.91
	FS18-S	31.47	47.20	39.70	3.81	9.61
	FS-C-BLUP	21.31	54.81	37.13	7.28	19.61
	FS-S-BLUP	23.92	51.46	38.89	6.59	16.96
MIC	MIC17-C	3.38	4.99	4.19	0.27	6.51
	MIC17-S	3.62	4.98	4.25	0.27	6.28
	MIC18-C	3.11	5.12	4.02	0.33	8.31
	MIC18-S	3.10	5.23	4.11	0.33	8.13
	MIC-C-BLUP	2.69	5.74	4.11	0.53	12.87
	MIC-S-BLUP	2.69	5.92	4.18	0.52	12.45
FU (%)	FU17-C	83.35	88.13	86.28	0.90	1.04
	FU17-S	84.40	88.65	87.07	0.81	0.93
	FU18-C	82.56	87.98	85.64	0.91	1.06
	FU18-S	81.75	86.55	84.97	0.81	0.95
	FU-C-BLUP	81.16	89.67	85.96	1.51	1.76
	FU-S-BLUP	81.40	88.46	86.02	1.34	1.56
FE (%)	FE17-C	6.62	7.11	6.89	0.10	1.48
	FE17-S	6.52	6.95	6.74	0.08	1.21
	FE18-C	6.65	7.07	6.88	0.07	1.08
	FE18-S	6.73	7.12	6.89	0.07	1.04
	FE-C-BLUP	6.52	7.18	6.89	0.13	1.88
	FE-S-BLUP	6.53	7.02	6.82	0.10	1.42

^a^17, 18 and BLUP indicated the phenotype data in 2017, 2018 and BLUP evaluated in the same environment, C: normal condition; S: salt condition

*FL* fiber length, *FS* fiber strength, *MIC* fiber micronaire, *FU* fiber uniformity, *FE* fiber elongation

### Genome-wide association studies

The GWAS was conducted for the five fiber quality traits with a multi-loci mixed linear model [13]. In total, 102 quantitative trait nucleotides (QTNs) on 26 chromosomes were identified as significantly associated with the traits (Additional file 7: Table S3). We define the flank 200 Kb regions of QTN as an initial QTL and merge the overlapped QTLs to obtain the final QTLs as described previously [14]. In total, 81 QTLs were detected 413 times under different environments and a best linear unbiased predictors (BLUP) by six multi-loci MLM models. Considering that it could be false positive in QTLs with less association frequency, we selected QTLs with more than three association times as stable QTLs for subsequent analysis (Additional file 8: Fig. S5). As a result, 34 stable QTLs were identified including nine high frequency associated QTLs with association times greater than 10 (Table 2 and Additional file 9: Table S4).

**Table 2:** Identification on stable QTL related to five fiber quality traits by multi-loci MLM model

Trait^a^	Environment	Number of QTL	QTL counts
FL	Normal	9	45
	Salt	9	41
	Total	11	86
FS	Normal	9	52
	Salt	8	49
	Total	11	101
MIC	Normal	10	35
	Salt	7	23
	Total	13	58
FU	Normal	2	12
	Salt	4	26
	Total	4	38
FE	Normal	10	36
	Salt	11	28
	Total	17	64
Total		34	347

^a^*FL* fiber length, *FS* fiber strength, *MIC* fiber micronaire, *FU* fiber uniformity, *FE* fiber elongation

**Fig. 3:**
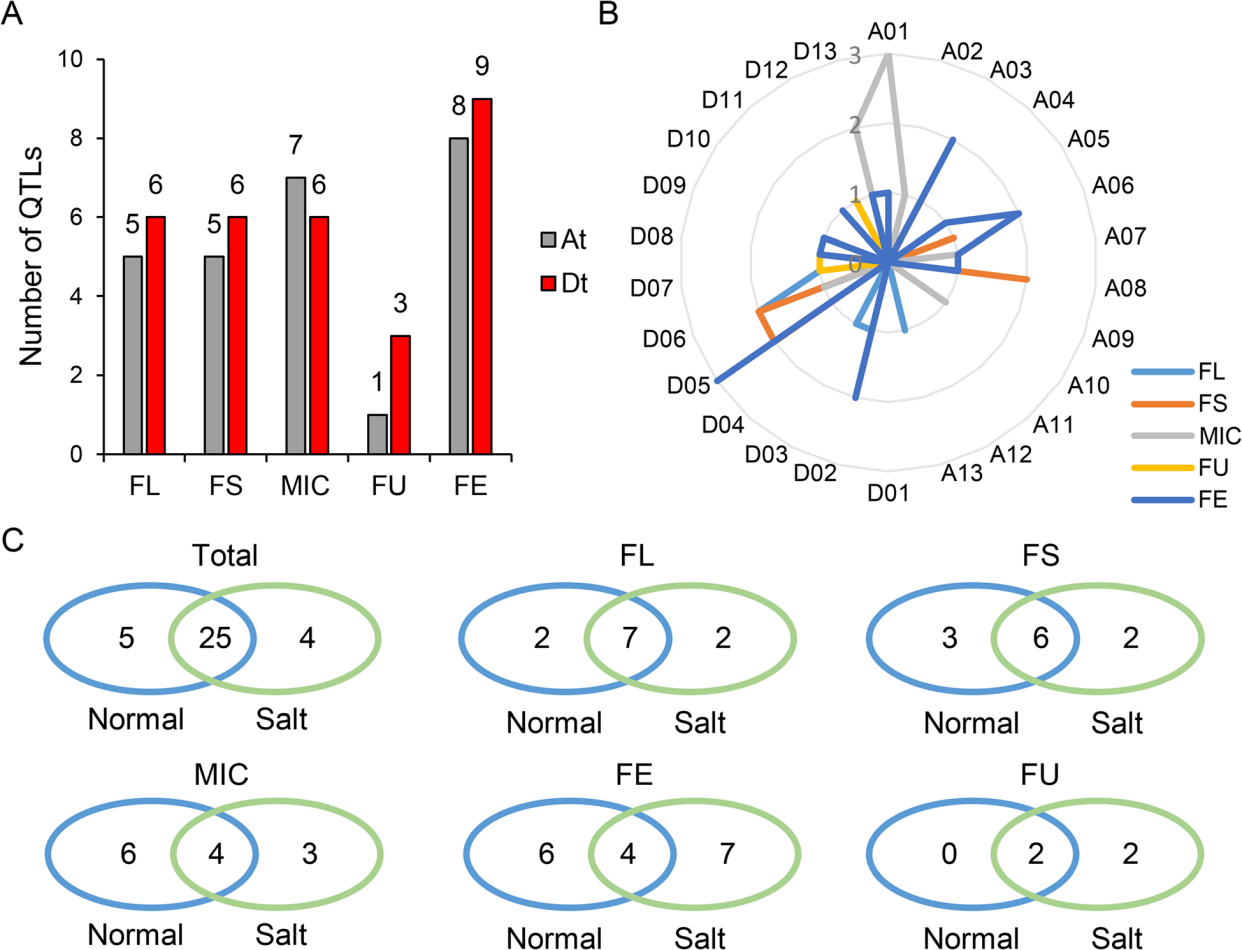
Identification of the stable QTLs associated with the five fiber quality traits in different conditions. **a**. Numbers of QTLs on At and Dt sub-genome. **b**. Numbers of QTLs on 26 chromosomes. **c**. Venn diagram of stable QTLs of five fiber quality traits under normal and salt environments

Due to the high correlation, the overlapped stable QTLs among the five traits were also detected (Additional file 9: Table S4). It showed that 18 stable QTLs for each of four traits (FL: 4, FS: 4, MIC: 5 and FE: 5) were specifically detected and 16 stable overlapped QTLs were detected in two or more traits. Of them, QTL TM6004 was detected to simultaneously associate with FL, FS, FE and FU, with the most association times of 75. There were also four QTLs were detected to be associated with three traits (Additional file 9: Table S4). These stable QTLs were widely distributed on At and Dt subgenome with a bit more loci of FL, FS, FE and FU on Dt subgenome (Fig. 3a). Chromosome distribution showed that most QTLs for MIC and FE were located on A01 and D05, respectively (Fig. 3b). In different environments, many overlapped QTLs were also detected under both normal and salt conditions (74% of total QTLs), especially for FL and FS with 64 and 55% overlapped QTLs, respectively (Fig. 3c), implying the high and stable heritability for most of fiber quality traits.

### Identification on candidate genes in QTLs

Potential candidate genes in these stable QTL regions were extracted based on the released *G. barbadense* Hai7124 genome [15]. In total, 998 candidate genes were identified in the stable QTL regions, with 302 of FL, 251 of FS, 373 of MIC, 84 of FU and 583 of FE (Additional file 10: Table S5). Considering the two environments, 118 and 95 genes were specifically identified associated with the fiber quality traits under normal and salt conditions, respectively, while 785 genes were simultaneously identified. GO analysis showed that the 95 genes specifically identified in salt conditions were enriched in “response to stimulus” (*P*-value 4.8 × 10^− 5^), and the 118 genes specifically identified in normal conditions were mainly involved in “cellular process”. We found the 785 candidate genes simultaneously identified under normal and salt conditions were enriched mostly in “cellular process” and “biological process”. Also, “xylem development”, “response to hormone” and “regulation of post-embryonic development” were also detected (Additional file 11: Table S6). According to tissues expression profile, we further identified 572 fiber expressed genes in these stable QTL regions, indicating they play roles in fiber development (Additional file 12: Table S7).

### Mining of key genes for fiber quality traits

**Fig. 4:**
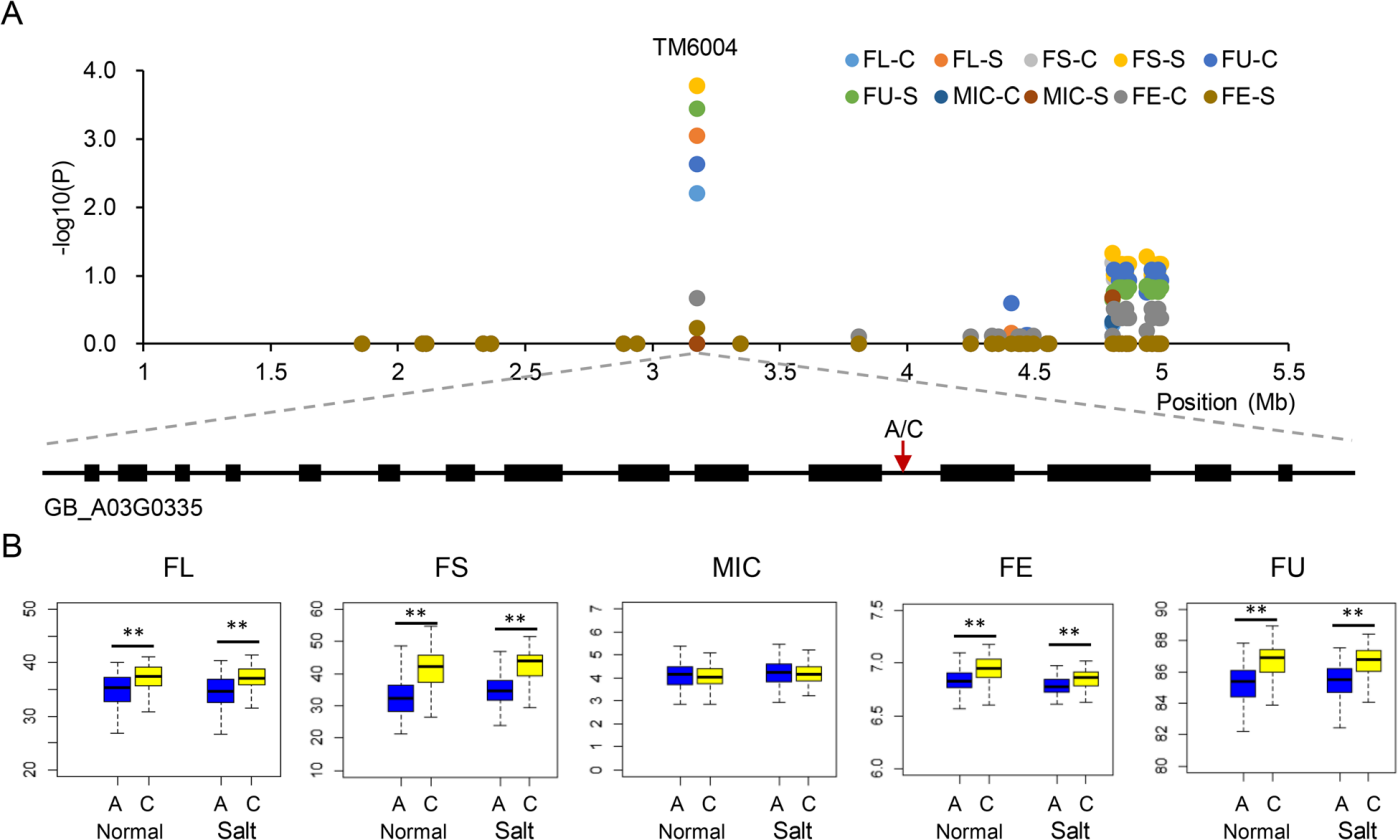
Contribution of QTL TM6004 to fiber quality traits. **a**. Manhattan plots of SNPs around TM6004 for the best linear unbiased prediction (BLUP) of five traits. The location of a QTN for A/C related to *GB_A03G0335*. **b**. Box plots for the phenotypic values of QTN TM6004

We further analyzed the nine high frequency associated QTLs for mining key candidate genes. The QTL TM6004, which had a QTN at 4,186,816 bp of the QTL region located on A03: 3,986,816 bp - 4,386,816 bp (Fig. 4a), was simultaneously associated with FE, FL, FS and FU with high frequency detected times, and explained more than 19.9% of the variation in fiber quality traits both in the normal and salt environments. Sequence analysis showed that the QTN of TM6004 (from A to C) was located in the intron region of the gene *GB_A03G0335* which encodes an E3 ubiquitin-protein ligase. Through a Student’s *t* test, we found that the FL, FS, FU and FE increased respectively by 6.9, 22.5, 1.5 and 1.2% with the C haplotype comparing with the A haplotype (Fig. 4b), implying that QTN TM6004 might play important roles in fiber development. Beside this, another candidate gene, *GB_A03G0342* which encodes a laccase 3, was also identified. Laccase enzymes play important roles in cell elongation, lignification and pigmentation in plants and could play crucial role in cotton fiber quality [16]. Another high frequency associated QTL TM68443, which located on D08: 21,500,627 bp-21,900,627 bp with SNP of A to G, was simultaneously associated with FL, FS and FU under the normal and salt environments. It showed that the FL, FS and FE increased by 8.1, 26.9 and 1.5% with the A haplotype comparing with the G haplotype, respectively (Additional file 13: Fig. S6). In the QTL region, a gene *GB_D08G1034* encodes an auxin canalization protein, was identified. The previous study showed that auxin transport played an important role in fiber development of upland cotton [14]. In addition, we also found five high frequency QTLs only associated with a single trait, including one associated with FL, two associated with MIC, and two associated with FE. In these QTLs, many of the genes were related to fiber development, such as *GB_D09G2489* encodes a Ca^2+^-binding protein 1 [17] and *GB_D09G2484* encodes a sodium hydrogen exchanger 2 [18], being important genes in fiber development under normal or salt environments.

## Discussion

Upland cotton (*G. hirsutum*) and sea island cotton (*G. barbadense*) are two fiber-producing tetraploid cultivated cotton species, with markedly superior fiber quality in sea island cotton. However, there are few studies on the genetic characteristics of sea island cotton in natural population. In this study, we used high quality 6303 SNPs to investigate the genetic diversity and population characteristics of 279 sea island cotton accessions. Compared with the previous studies based on other molecular markers, such as ISSR [19], SRAP [20] and SSR [21], the number of SNP markers were greatly increased in present study. The mean PIC value of the 279 accessions showed that the PIC of At sub-genome was higher than that of Dt sub-genome. Phylogenetic tree and PCA analysis supported that the 279 accessions could be clustered into three groups, which was similar to previous studies based on SSR markers [21, 22]. Modern cultivated sea island cotton mainly includes three ecotypes: Egyptian type, American type and Middle-Asia type [22]. Of the 39 introduced landraces, Group1 contained three introduced accessions mainly from Egypt, of them, an original sea island cotton landrace, Menoufi, was also clustered in Group1. Group2 and Group3 were involved in widely introduced accessions from Egypt, USA and Former Soviet Union, with 31 in Group2 and five in Group3, implying that Group1 from original ecotype had the highest differentiation compared with the other two groups. At present, the genetic basis of modern cultivated sea island cotton is relatively narrow. Based on genetic diversity of three groups, the accessions from different groups can be crossed to pyramid multi-loci related to target traits for genetic improvement in future sea island cotton breeding.

Salinity is considered to be one of the most significant environmental factors limiting the growth and development of crop plants. In China, sea island cotton grows mainly in Xinjiang ecological area, however, most of the cultivated land is saline alkali in this region. Few studies were reported in stress condition for sea island cotton. Here, using sea island cotton natural population, we selected two different field environments, normal and saline alkali soil, to investigate systematically the fiber quality traits over 2 years. With phenotypic analysis, we found that FL and FE were significantly decreased in salt conditions, while FS and MIC were increased. In addition, no difference of FU was detected in the two environments. Of these changes, FS increased significantly in salt conditions with the largest variation compared with other four traits. High correlation among five fiber quality traits further supported the results. Consistent with the previous studies [11, 23], fiber length was decreased under salt stress, which required the sea island cotton varieties with improvement on fiber qualities, especially in fiber length, for planting in saline alkali condition.

Via GWAS analysis, a total of 34 stable QTLs, including nine high frequency associated loci, were identified to be related to five fiber quality traits. About half of these QTLs were associated with multiple traits, which was consistent with a high correlation among five fiber quality traits by phenotypic analysis. We found that four QTLs, TM27446_TM27440, TM33841_TM33859, TM58446 and TM68443, overlapped with the fiber quality traits associated QTLs of sea island cotton, reported recently by re-sequencing analysis [24]. Of them, TM68443 was identified as a high frequency associated QTL for FL, FS and FU in present study, and A haplotype of this QTL could significantly increase FL, FS and FE of sea island cotton. In addition, most QTLs, especially for FL and FS, were both detected in normal and salt conditions, indicating the high heritability of fiber quality traits in different environments. Besides these, we detected a fraction of QTLs associated with fiber quality specifically in salt environment and found the genes located in the QTLs were enriched in “response to stimulus”, which might play important role for fiber improvement in saline alkali condition. We also found that the candidate genes simultaneously identified under normal and salt conditions were enriched in “xylem development”, implying xylem participating in fiber development. Xylogen in the cell walls of cotton fibers effectively provides mechanical strength, while its functional role in fiber quality of sea island cotton remains to be explored.

To further mine the key genes affecting fiber qualities, we analyzed the high frequency QTLs detected more than 10 association times. Interestingly, seven of the total nine high frequency QTLs were detected both under normal and salt conditions. The QTL TM6004, which explained more than 21.6% of the variation in fiber quality traits. In this QTL region, the gene *GB_A03G0335* which encodes a E3 ubiquitin-protein ligase, with a QTN variation from A to C in its intron region, showed that the FL, FS, FU and FE increased significantly by 6.9, 22.5, 1.5 and 1.2% with the C haplotype comparing with the A haplotype, respectively. Protein ubiquitination plays key roles in multiple plant developmental stages and several abiotic stress responses [25]. Ubiquitin-protein ligase has also reported to be involved in several biological processes in cotton. Overexpression an ubiquitin ligase gene, *GhHUB2*, increased fiber length and SCW thickness, while RNAi knockdown of *GhHUB2* resulted in shortened fibers and thinner cell walls in cotton, which was involved in the ubiquitin-26S proteasome pathway [26]. The ubiquitin E3 ligase RHA2b promoted degradation of MYB30 [27], while MYB transcription factors were widely reported to be related to fiber development [28, 29]. Furthermore, E3 ubiquitin ligase was also reported to be involved in stress tolerance in cotton, such as *Verticillium dahlia* [30], drought [31] and salt tolerance [32]. Overexpression of a rice SUMO E3 ligase gene *OsSIZ1* in cotton enhanced drought and heat tolerance, and substantially improved fiber yields in the field under reduced irrigation and rainfed farming conditions [33]. Based on transcription pattern in various vegetative and fiber development tissues in *G. barbadense* cv. Hai7124, we also found multiple types of transcripts of *GB_A03G0335*, implying that the gene *GB_A03G0335* may play an important role in cotton fiber development and other stress tolerance. However, the molecular mechanism remains to be confirmed in further study.

## Conclusions

It is of great significance to explore the genetic characteristics and screen the key elite loci in sea island cotton for fiber quality improvement. In this study, we systematically reported genetic diversity of a set of sea island cotton natural population, and developed the phenotypic and GWAS analysis of five fiber quality traits under normal and salt environments. We found that the sea island cotton accessions were clustered into three groups with wide genetic diversity. There were high correlation of five fiber quality traits whether among traits in same environment or the same trait in different environments. Based on SNPs and GWAS analysis, a total of 34 stable QTLs were identified for the five fiber quality traits with 25 detected simultaneously under normal and salt environments. Haplotype analysis found that *GB_A03G0335* encoding an E3 ubiquitin-protein ligase, play a crucial role in fiber quality and was significantly correlated with FL, FS, FU and FE. These findings will help us understand the genetic diversity of sea island cotton and contribute to cotton fiber quality improvement in breeding practice.

## Methods

### Plant materials

A total of 279 sea island cotton accessions, with 240 cultivars/lines collected from China and 39 introduced from abroad, were used for genotyping and population characteristic analysis, and 249 accessions were used for fiber phenotype analysis (Additional file 1: Table S1). Of these accessions, 11 landraces (E24–3389, Giza 36, Giza 45, L-3398, Mi 10, Pima, Pima S-3, Termez 16, Yinzi 6022, 504-N, 9899И) were collected from the National Medium-term Gene Bank of Cotton in China, and other 268 accessions were collected and preserved by Nanjing Agricultural University and Xinjiang Agricultural University, China. All accessions were authorized for planting and investigating by Nanjing Agricultural University, Xinjiang Agricultural University and the National Medium-term Gene Bank of Cotton in China.

### Phenotype investigation and data analysis

In 2017 and 2018, the 279 sea island cotton accessions were planted in two natural environments in Xinjiang Agricultural University Experimental Station. All necessary permits for the field evaluations of these accessions were authorized by Xinjiang Agricultural University, China. To assess soil salt concentration in the fields, soil samples were collected at 15–30 cm depth with nine sampling points for each environment before cotton planting, and were analyzed for electrical conductivity (EC) using the method described by Singh et al. (2017) [34]. The average soil EC was 0.5 and 2.7 dS/m in the two natural fields, and marked as normal and salt environments, respectively. Each accession was grown in two rows with a 3 m row length and 0.13 m between plants with two replicate plots in each environment. We collected 30 well developed open boll samples from the middle branches of each accession for fiber qualities detection. Totally, five fiber quality traits, FL, FS, MIC, FU and FE, were measured using high volume fiber tester HFT9000 (ZXYQ09–2) by the Center of Cotton Fiber Quality Inspection and Testing, Ministry of Agriculture and Rural Affairs. Raw phenotypic data were also normalized across replicates, years, and environments using BLUP based on a mixed linear model in R package ‘lme4’ [35]. Descriptive statistics of phenotype data are calculated through Excel software. Paired-samples *t*-tests and correlation analysis of phenotype data among different environments and traits were performed using SPSS software. Due to insufficient seedlings for phenotype investigation in 30 accessions, we used the phenotypic data of fiber quality traits from 249 accessions for further GWAS analysis.

### SNP genotyping

For SNP genotyping, young leaves were collected from the 279 cotton accessions (a total of 288 samples, with eight collected repeatedly from different origins) and genomic DNA was extracted using CTAB method described by Paterson et al. (1993) [36]. A CottonSNP80K array containing 77,774 SNPs, was used to genotype the accessions. Qualified DNA was hybridized on the CottonSNP80K array and genotyped with the protocols described by Cai et al. (2017) [8]. The initial SNP data set was filtered with a calling rate < 0.8 and MAF < 0.01. In addition, in order to determine the physical location of SNP on the reference genome, we mapped the probe sequences back to the Hai7124 genome [15] and the probe sequences mapped to multiple loci were further filtered. The finally obtained high quality data set was used for subsequent genetic and GWAS analysis.

### Population characteristics

The SNP data format was adjusted by self-written shell script and used to conduct similarity analysis of 288 cotton accessions by PLINK V1.90 software [36]. For the construction of phylogenetic tree, a neighbor-joining method was used by Tassel 5.0 [37], and visually edited by FigTree software (http://tree.bio.ed.ac.uk/software/figtree/). Principal component analysis (PCA) was employed using Tassel 5.0 software and plot was drawn by R package “ggplot2”. Admixture 1.3 software was applied to population structure analysis with K = 3 [38]. Nucleotide diversity (π) and population divergence (F*st*) between pairwise groups of sea island cotton accessions were calculated using Vcftools software [39].

### GWAS analysis

The GWAS analysis was conducted using a multi-locus random-SNP-effect mixed linear model (mrMLM) [13] in R package “mrMLM”. For the associated SNPs filtering in the first step of the model, the following parameters were set: Critical *P*-value in rMLM: 0.001; Search radius of candidate gene (Kb): 100; and Critical LOD score in mrMLM: 3. Meanwhile, the Q + K model was also set with a population structure (Q) matrix calculated by admixture 1.3 and a kinship (K) matrix calculated by R package “mrMLM”. The Manhattan plot of associated QTN was performed by R software using the BLUP values of five traits in two environments.

### Identification of candidate genes

We define the flank 200 Kb regions of QTN as the same QTL, and merge the overlapped QTLs to confirm the number of QTL. Putative candidate genes were identified in the QTL regions from the reference genome by self-written shell scripts. To determine the function of the candidate genes, Gene ontology (GO) analysis was implemented using AgriGO V2.0 with the SEA method [40]. The transcriptome profiles of various tissues and organs from Hai7124 were downloaded from NCBI Sequence Read Archive collection PRJNA490626 [15] and analyzed for mining candidate genes during fiber development. When FPKM (the fragments per kilobase of exon model per million mapped reads) of a gene was greater than 3 in ovules and fibers at 0–25 day post anthesis (DPA), the gene was considered to be expressed during fiber development.

## Supplementary information

**Additional file 1 Table S1.** Information on 279 sea island cotton accessions used in this study.

**Additional file 2 Figure S1.** Call frequency distribution of total SNPs genotyped by CottonSNP80K array.

**Additional file 3 Figure S2.** Distribution of SNP number on each chromosome.

**Additional file 4 Table S2.** Information on high quality SNPs genotyped by CottonSNP80K array.

**Additional file 5 Figure S3.** Density distributions of five fiber quality traits in sea island cotton natural population.

**Additional file 6 Figure S4.** Correlation analysis of five fiber quality traits under different conditions. The number in these boxes indicated correlation coefficient (R value). *, **, and *** indicated *P* value at the 0.05, 0.01 and 0.001 levels, respectively.

**Additional file 7 Table S3.** Stable QTLs of five fiber quality traits identified by multi-loci mixed linear model.

**Additional file 8 Figure S5.** Distribution on detected times for 81 associated QTLs of five traits, respectively. The x-axis represents the detected times; y-axis represents the number of QTLs.

**Additional file 9 Table S4.** Information on the associated times and corresponding traits for stable QTLs.

**Additional file 10 Table S5.** Candidate genes associated with fiber quality traits within QTL regions.

**Additional file 11 Table S6.** GO analysis of genes specifically and simultaneously associated under normal and salt environments, respectively.

**Additional file 12 Table S7.** Expression profile of genes located in QTL regions related to fiber qualities.

**Additional file 13 Figure S6.** Box plots for the phenotypic values of QTN TM68443.

## Data Availability

RNA-Seq data in this study have been deposited at the National Center of Biotechnology Information (NCBI, http://www.ncbi.nlm.nih.gov/) under the accessions PRJNA490626.

## Abbreviations

BLUP: The best linear unbiased prediction;
CV: Coefficients of variation
EC: Electrical conductivity
FE: Fiber elongation
FL: Fiber length
MIC: Fiber micronaire
FS: Fiber strength
FU: Fiber uniformity
GO: Gene Ontology
GWAS: Genome-wide association study
MAS: Marker assisted selection
MLM: Multi-loci mixed linear model
PIC: Polymorphism information content
QTLs: Quantitative trait loci
QTNs: Quantitative trait nucleotides
SLAF-seq: Specific-locus amplified fragment sequencing
SNPs: Single nucleotide polymorphisms
