# Supplementary figures and images for: Genome-wide association analysis reveals loci and candidate genes involved in fiber quality traits in sea island cotton (Gossypium barbadense)
Source: BMC Plant Biol. 2020 Jun 22;20:289. doi: 10.1186/s12870-020-02502-4 (PMC7310526; doi:10.1186/s12870-020-02502-4)

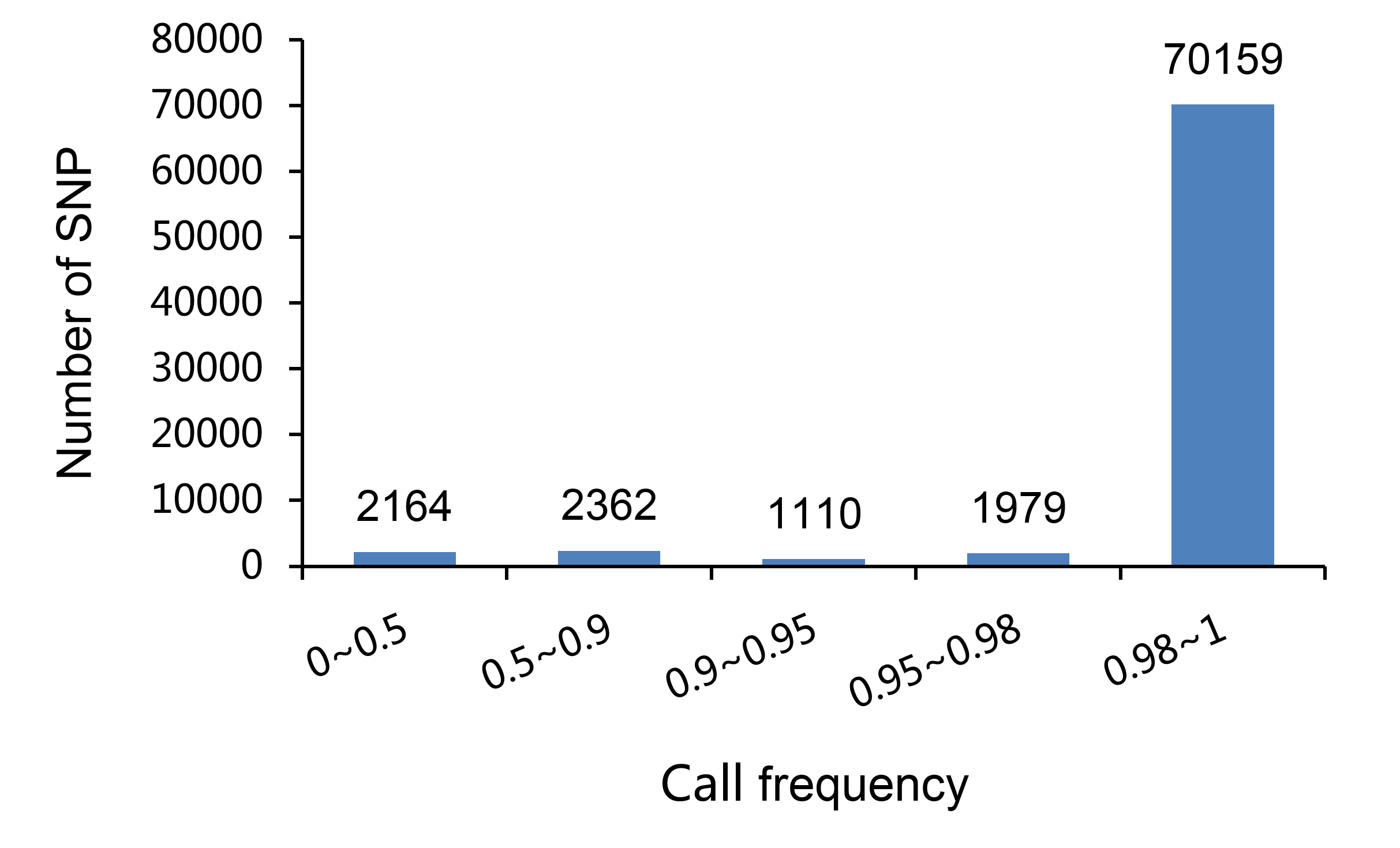

Supplement: Supplementary file 2 — Additional file 2 Figure S1. Call frequency distribution of total SNPs genotyped by CottonSNP80K array. [file 12870_2020_2502_MOESM2_ESM.tif]

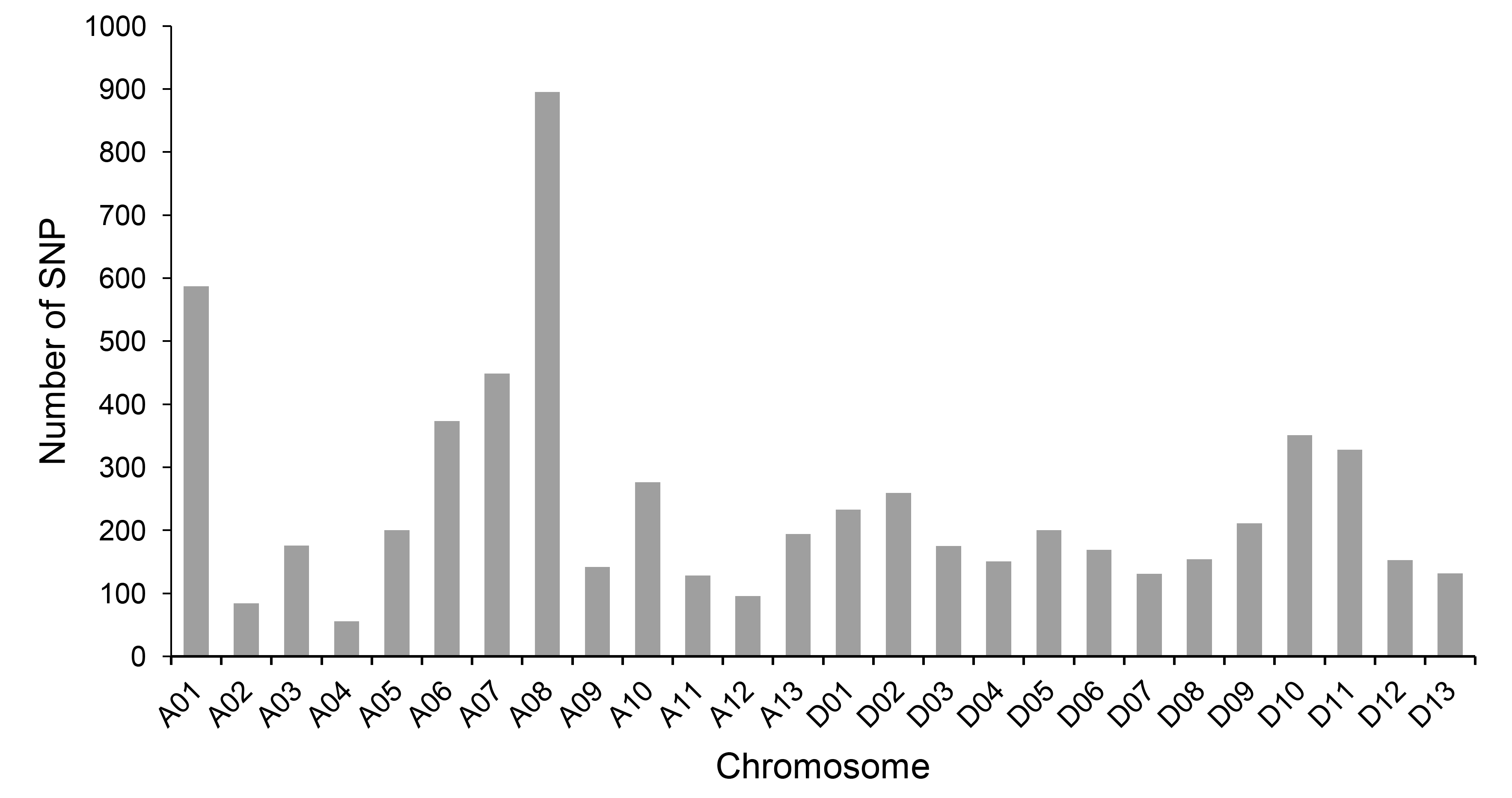

Supplement: Supplementary file 3 — Additional file 3 Figure S2. Distribution of SNP number on each chromosome. [file 12870_2020_2502_MOESM3_ESM.tif]

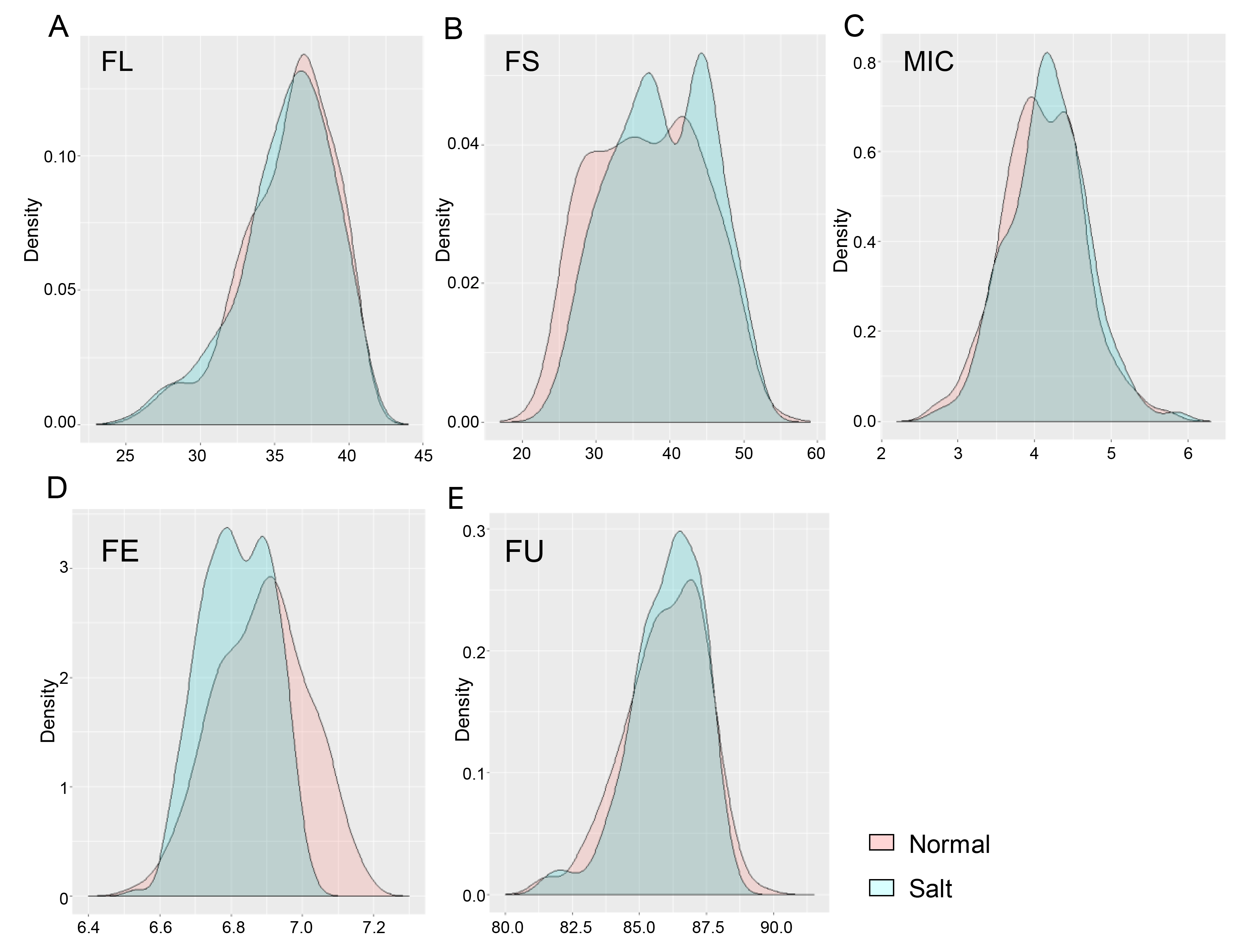

Supplement: Supplementary file 5 — Additional file 5 Figure S3. Density distributions of five fiber quality traits in sea island cotton natural population. [file 12870_2020_2502_MOESM5_ESM.tif]

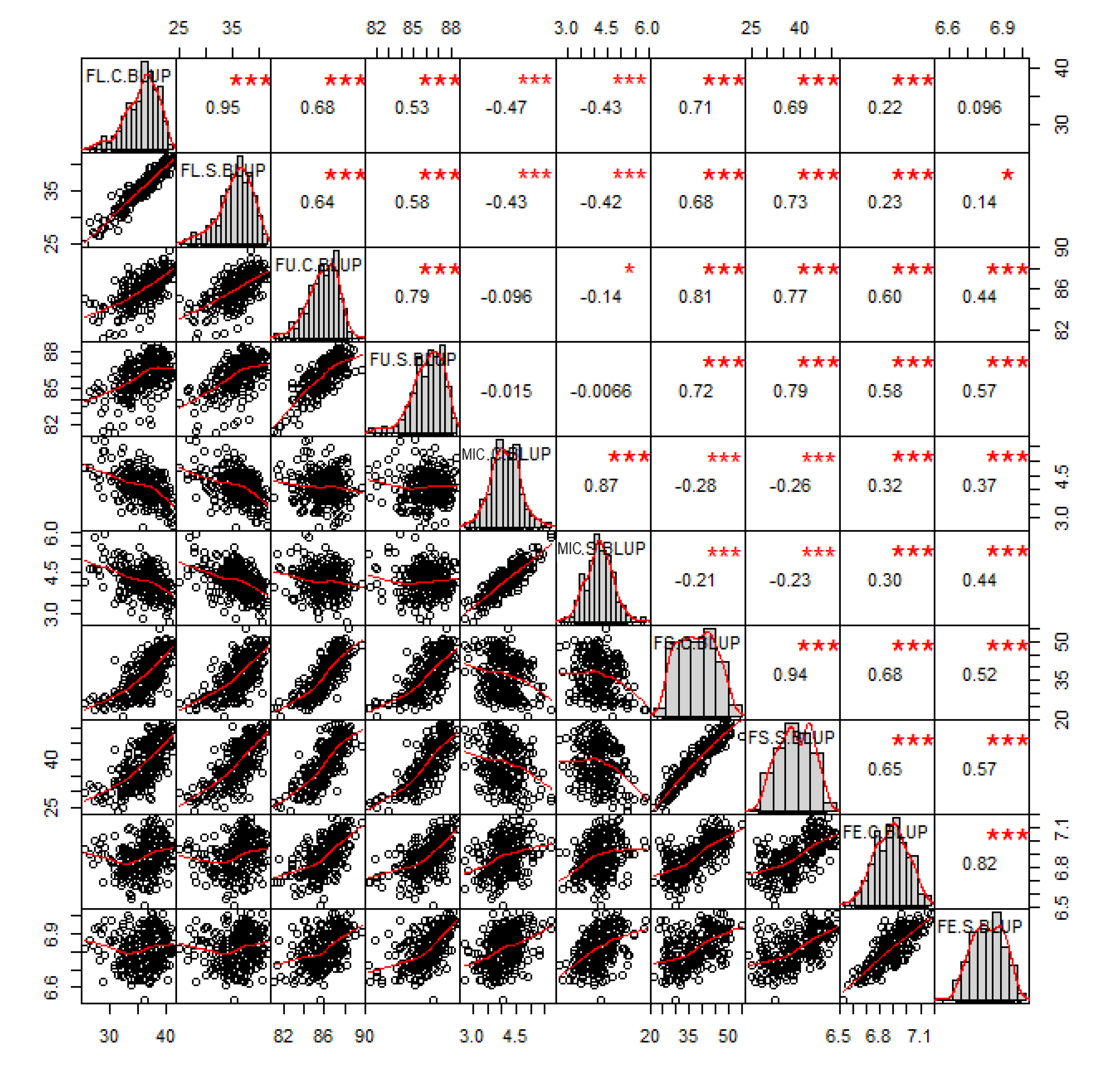

Supplement: Supplementary file 6 — Additional file 6 Figure S4. Correlation analysis of five fiber quality traits under different conditions. The number in these boxes indicated correlation coefficient (R value). *, **, and *** indicated P value at the 0.05, 0.01 and 0.001 levels, respectively. [file 12870_2020_2502_MOESM6_ESM.tif]

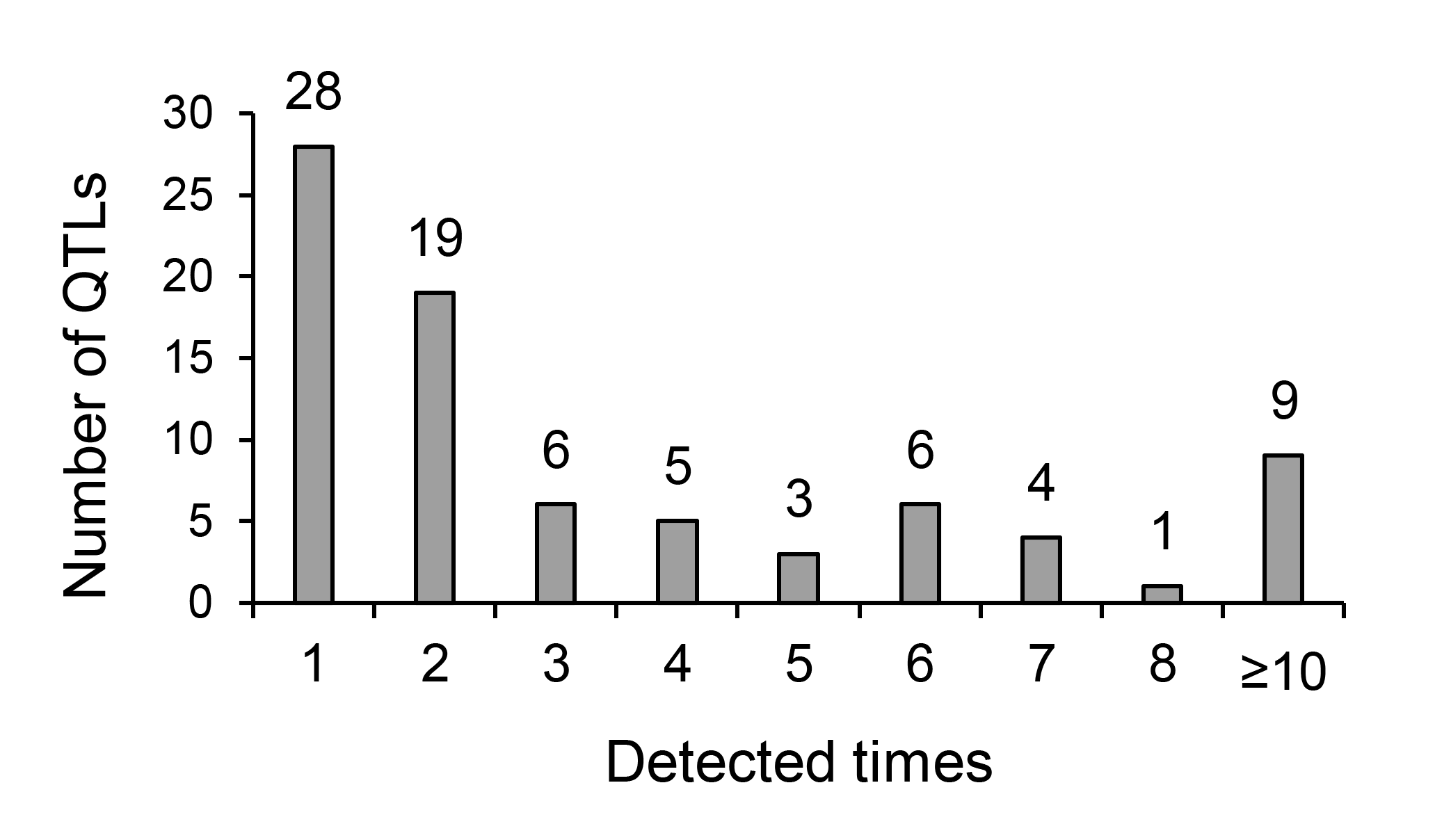

Supplement: Supplementary file 8 — Additional file 8 Figure S5. Distribution on detected times for 81 associated QTLs of five traits, respectively. The x-axis represents the detected times; y-axis represents the number of QTLs. [file 12870_2020_2502_MOESM8_ESM.tif]

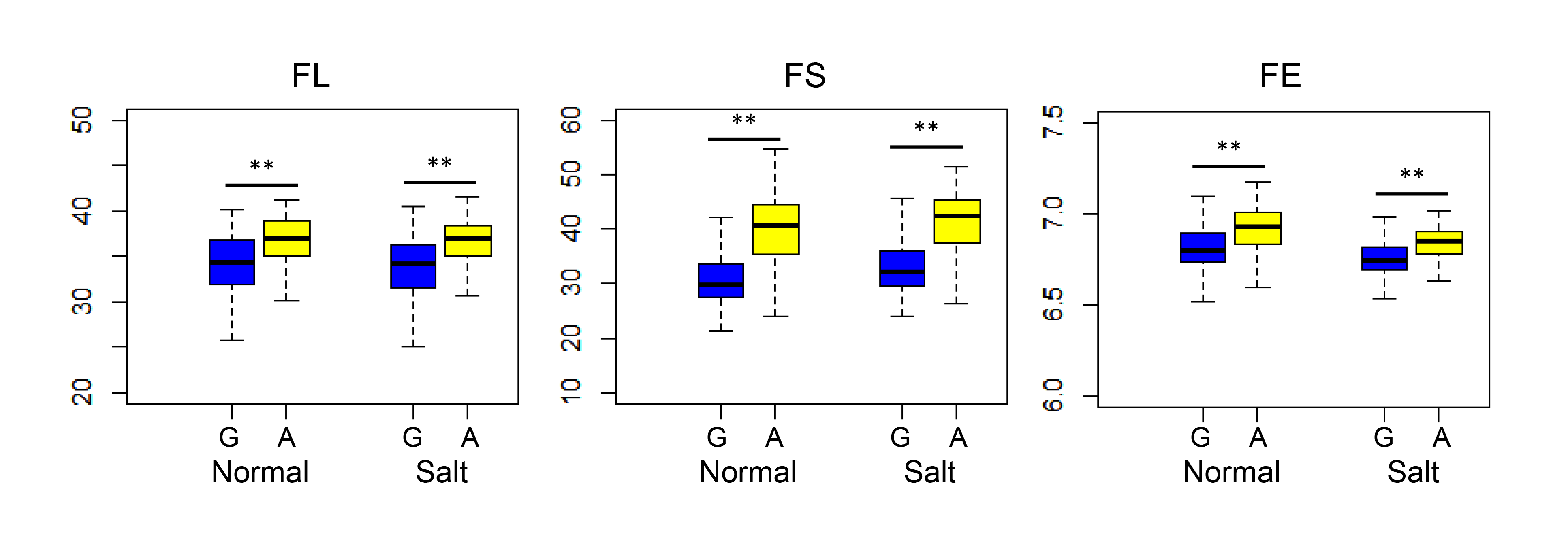

Supplement: Supplementary file 13 — Additional file 13 Figure S6. Box plots for the phenotypic values of QTN TM68443. [file 12870_2020_2502_MOESM13_ESM.tif]
